# Supplementary material for: Mutations in PCYT2 disrupt etherlipid biosynthesis and cause a complex hereditary spastic paraplegia
Source: Brain. 2019 Oct 22;142(11):3382–97. doi: 10.1093/brain/awz291 (PMC6821184; doi:10.1093/brain/awz291)
Supplement: awz291_Supplementary_Data [file awz291_supplementary_data.zip › awz291-Suppl_data/Supplementary_Data4.zip!/Supplementary information 4 - plasma lipidomics.zip!/Plasma lipidomics/Control vs Patient1/Statistical plots and data/S1P species/Boxplots.pdf]

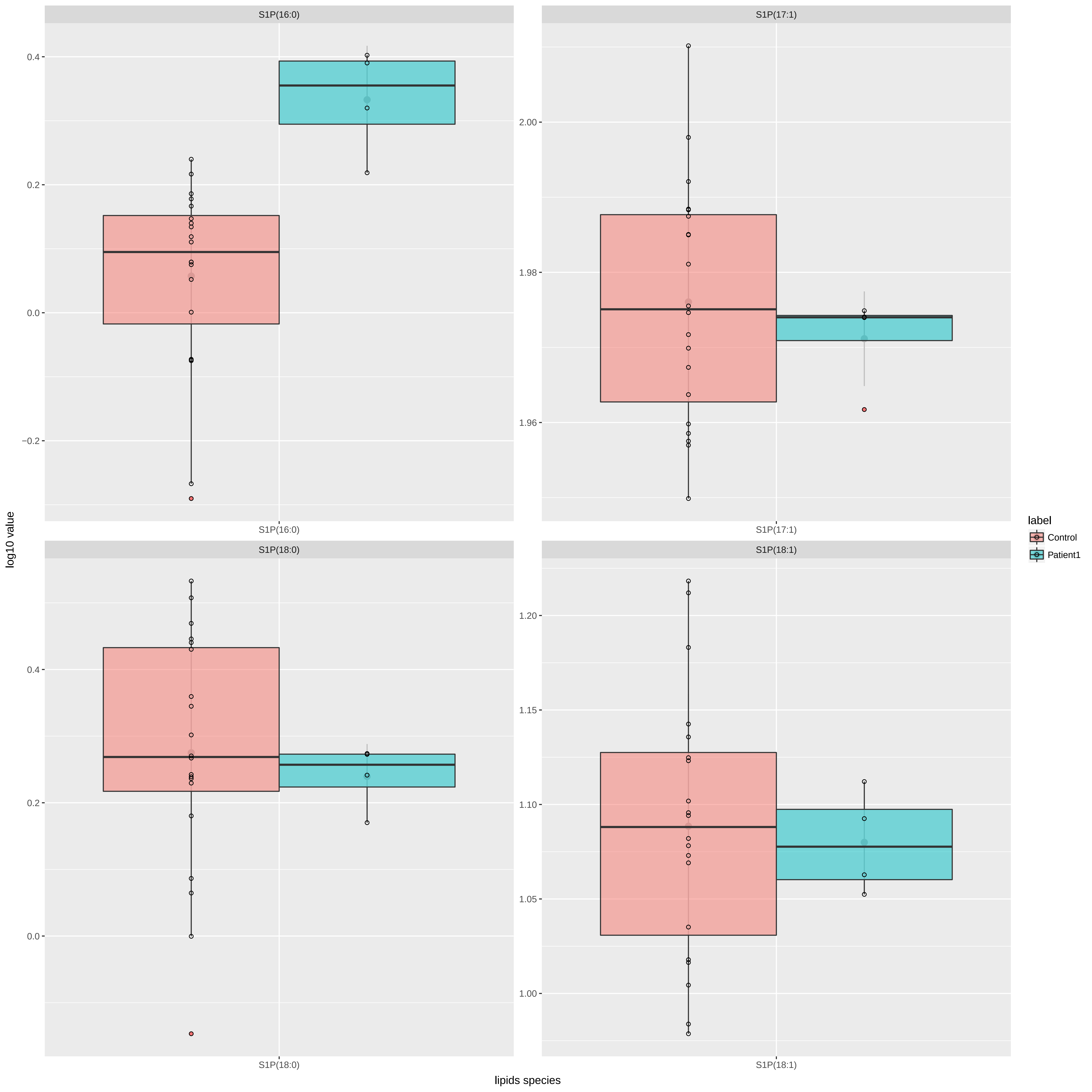

S1P(16:0)
S1P(17:1)
S1P(18:0)
S1P(18:1)
0.4
0.2
0.0
−0.2
2.00
1.98
1.96
1.20
1.15
1.10
1.05
1.00
log10 value
lipids species
label
Control
Patient1
